# Clinicopathological Characteristics and Outcomes of Lupus Nephritis Patients With Thrombocytopenia: A Single‐Center Retrospective Study

**DOI:** 10.1002/iid3.70179

**Published:** 2025-03-19

**Authors:** Hui Diao, Yuting Fan, Di Kang, Zhiqing Chen, Yuewen Lu, Xiamin Huang, Xi Xia, Wei Chen

**Affiliations:** ^1^ Department of Nephrology The First Affiliated Hospital, Sun Yat‐Sen University Guangzhou China; ^2^ NHC Key Laboratory of Clinical Nephrology (Sun Yat‐Sen University) and Guangdong Provincial Key Laboratory of Nephrology Guangzhou China

**Keywords:** lupus nephritis, SLE‐DAI, thrombocytopenia

## Abstract

**Objectives:**

The objective of this study is to analyze and summarize the clinical characteristics and prognosis of lupus nephritis (LN) patients with thrombocytopenia and to improve the cognition of the disease.

**Methods:**

896 LN patients were enrolled in this study and their clinical and pathological data were collected and analyzed. The primary end point was mortality. The secondary end point was adverse renal outcomes, defined as doubling of the baseline serum creatinine or end‐stage renal diseases. Cox regression model was used to analyze the risk factors of mortality or renal events in LN with and without thrombocytopenia.

**Results:**

Among 896 LN patients, 70 (7.8%) were diagnosed with thrombocytopenia. LN patients with thrombocytopenia had lower estimated glomerular filtration rate (eGFR) and higher systemic lupus erythematosus disease activity index (SLE‐DAI), proportion of anemia, leukopenia, hypocomplementemia, and positive anti‐cardiolipin antibodies, compared to those without thrombocytopenia. LN patients with thrombocytopenia had higher scores of activity index and more activity features (endocapillary hypercellularity, medullary loop necrosis) on kidney biopsy. There was no significant difference in patient survival and renal survival between LN patients with and without thrombocytopenia. Anemia was a risk factor for death in LN patients with thrombocytopenia and lower eGFR was a risk factor for adverse renal outcomes.

**Conclusions:**

LN patients with thrombocytopenia showed higher disease activity, more anti‐cardiolipin antibody positivity and a higher activity index in kidney biopsy, but the prognosis was similar compared with those without thrombocytopenia. Anemia was a risk factor for death in LN patients with thrombocytopenia.

## Summary

- LN patients with thrombocytopenia showed a higher SLE disease activity index.
- The prognosis was similar between LN patients with and without thrombocytopenia.
- Anemia was a risk factor for death in LN patients with thrombocytopenia.

## Graphical abstract

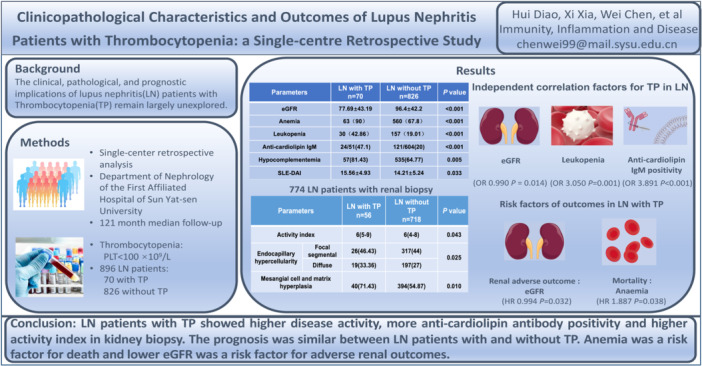

## 1 Introduction

The clinical presentation of systemic lupus erythematosus (SLE) can involve one or more organs with the skin, joints, hematological systems, and kidneys being major sites of organ disease in Chinese SLE patients. Approximately 50% of patients will develop LN [1]. LN is one of the most common and serious manifestations of SLE, contributing significantly to morbidity and mortality of SLE [2]. It has been reported that thrombocytopenia is a common hematology disorder in SLE patients and has been included in the diagnostic criteria for SLE [3, 4]. Several studies have reported that the prevalence rates of thrombocytopenia in patients with SLE ranging from 10% to 40% [5, 6, 7, 8, 9, 10, 11]. In the CSTAP cohort, which included 2104 Chinese SLE patients, 342 individuals (16.3%) presented with thrombocytopenia at the time of initial assessment. The present investigation revealed that SLE patients with thrombocytopenia displayed more severe clinical manifestations (myositis, pleuritis, and fever), increased SLE‐DAI scores, higher frequencies of nephritis, and reduced survival rates [7]. A retrospective investigation involving a total of 330 patients diagnosed with SLE found that 73 exhibited thrombocytopenia, and individuals with thrombocytopenia displayed higher frequencies of nephritis compared to the control group [6]. These studies demonstrate a positive correlation between the presence of thrombocytopenia in SLE patients and the occurrence of LN. Another study investigated the platelet counts in a total of 20 LN patients, and 15 of the 20 LN patients exhibiting one or more occurrences of thrombocytopenia [12]. Nevertheless, the clinical, pathological, and prognostic implications of LN patients with thrombocytopenia remain largely unexplored.

The objective of this investigation was to examine the clinical and renal pathological features, as well as the outcomes, of individuals exhibiting thrombocytopenia within a Chinese LN cohort. The prognostic implications of thrombocytopenia on renal outcomes remain contentious. Our study's robust sample size will provide valuable insights for clinical practitioners.

## 2 Materials and Methods

### 2.1 Patients

This study extracted clinical data from the database of LN (http://ln.medidata.cn) in the Department of Nephrology of the First Affiliated Hospital of Sun Yat‐sen University (January 1996–December 2011). The clinical data of 896 LN patients were included, of which 774 LN patients underwent renal biopsy. The deadline for follow‐up investigation was set for February 1, 2023. All patients included in this study met the SLE diagnostic criteria revised by the American Society of Rheumatology in 1997 [4]. All patients provided informed consent before being enrolled in the registration system. The exclusion criteria for this study are as follows: (1) The age is less than 14 years old; (2) Lack of complete clinical data; (3) End‐stage renal disease (ESRD) at the time of admission, with an estimated glomerular filtration rate (eGFR) of less than 15 mL/min/1.73 m^2^ or dialysis for more than 3 months or having undergone renal transplantation; (4) Drug‐induced; (5) Primary hematological disorders such as leukemia, lymphoma, myeloma, myelodysplastic syndrome, aplastic anemia, thalassemia, sickle cell disease, and hemophilia, etc.); (6) infection‐induced; (7) hypersplenism‐induced; (8) Malignant tumors; (9) other causes. Supporting Information Figure S1 shows the recruitment and exclusion flowchart.

This research protocol complies with the provisions of the Helsinki Declaration (revised in 2013). The Ethics Committee of the First Affiliated Hospital of Sun Yat‐sen University reviewed and approved the research plan (Ethical review NO. [2016]215).

### 2.2 Definition

Thrombocytopenia: platelet count is less than 100 × 10^9^/L. Anemia: the hemoglobin concentration of male patients is less than 120 g/L, and that of female patients is lower than 110 g/L (based on the Chinese clinical internal medicine and diagnostics) [13]. Leukopenia: the concentration of white blood cells is less than 4 × 10^9^/L. Hypocomplementemia: the concentration of complement C3 is less than 0.79 g/L. Proteinuria was calculated from a 24‐h urine collection. The primary end point was death, and the secondary end point was renal outcome, including doubling of serum creatinine (doubling of serum creatinine compared with baseline) or ESRD (eGFR < 15 mL/min/1.73 m^2^, dialysis for more than 3 months or had renal transplantation) [14, 15, 16]. eGFR was calculated using the Epidemiological Collaborative Equation of Chronic Renal Disease (CKD‐EPI) [17].

### 2.3 Data Collection and Evaluation

Essential patient information and clinical data such as: age, sex, fever, rash, photosensitivity, alopecia, mucosal ulcer, joint swelling and pain, and CKD stage were obtained from the original medical records. The included platelet data were collected at the time of admission. Thrombocytopenia was included at the start of the study. The laboratory examination data includes hemoglobin, red blood cells, white blood cells, lymphocytes, neutrophils, 24‐h proteinuria, serum creatinine, urea nitrogen, eGFR, serum albumin, microscopic red blood cells, microscopic white blood cells, microscopic granular tube type, etc. Serum antinuclear antibody (ANA), anti‐double‐stranded DNA (dsDNA) antibody, anti‐sm antibody, anti‐Sjögren syndrome antigen A (SSA) antibody, anti‐Sjögren syndrome antigen B (SSB) antibody, anti‐ribonucleoprotein (RNP) antibody, anti‐nucleosome antibody (AnuA), anti‐centromere antibody, complement C3 and C4, hypocomplementemia, anti‐cardiolipin IgM positivity and anti‐cardiolipin IgG positivity were measured in all patients at local laboratories. The SLE disease activity index (SLE‐DAI) was used to evaluate clinical disease activity [18]. The pathological results of renal biopsy tissues were reported according to ISN/RPS 2003 grading [19]. The treatments include glucocorticoid + cyclophosphamide (GC+CTX), glucocorticoid + mycophenolate mofetil (GC + MMF), glucocorticoid + calcineurin inhibitor (GC + CNI), and others.

### 2.4 Statistical Analysis

The quantitative variables with a normal distribution are expressed as mean ± standard deviation (mean ± sd) and compared using *t*‐test. The quantitative variables with a non‐normal distribution are represented by the median and the interquartile difference (IQR) and compared using Wilcoxon's rank sum test. The categorical variables are expressed as frequency (percentage) and compared using the chi‐square test or Wilcoxon rank sum test. Multivariate binary logistic regression analysis was performed for the variables with *p* < 0.05 in univariate analysis and important variables for clinical concern. Kaplan–Meier survival analysis curve and multivariate Cox regression were used to compare the mortality and renal outcome between LN patients with thrombocytopenic and without thrombocytopenic. All data were analyzed using SPSS 26.0 statistical software (SPSS, Chicago, USA). Results were expressed as a hazard ratio (HR) with 95% confidence interval (CI). Two side *p* < 0.05 was considered statistically significant, and has already been bolded in the tables.

## 3 Results

### 3.1 Clinical Characteristics of LN Patients With Thrombocytopenia and Without Thrombocytopenia

As shown in Table 1, this study included 896 patients with LN, 70 of whom (7.8%) conformed to the criteria for thrombocytopenia. The mean (SD) platelet count in thrombocytopenia group was 71 ± 21 × 10^9^/L. The average age at diagnosis in the thrombocytopenia group was 27 ± 10 years old, which was younger than that of the control group (*p* = 0.046).

**Table 1:** Comparison of clinical and laboratory characteristics in LN patients between the two groups.

Parameters	Total	With thrombocytopenia	Without thrombocytopenia	*p* values
Number of patients (N)	896	70	826	
Platelet count (1000/L)	203.13 ± 81.063	71.86 ± 21.172	214.26 ± 74.197	
Male/female (*n*/*n*)	153/896	13/57	140/686	0.729
Age (years), m ± sd	30.31 ± 10.98	27.71 ± 10.56	30.52 ± 11.94	**0.046**
Fever (*n*, %)	275 (30.69)	24 (34.29)	251 (30.39)	0.497
Malar rash (*n*, %)	389 (43.42)	34 (48.57)	355 (42.98)	0.365
Photosensitivity (*n*, %)	96 (10.71)	7 (10)	89 (10.77)	0.841
Alopecia (*n*, %)	160 (17.86)	10 (14.29)	150 (18.16)	0.416
Oral ulcer (*n*, %)	75 (8.37)	12 (17.14)	63 (7.63)	**0.006**
Arthralgia (*n*, %)	352 (39.29)	30 (42.86)	322 (38.98)	0.524
eGFR (mL/min/1.73 m^2^), m ± sd	79.94 ± 41.77	77.69 ± 43.19	96.4 ± 42.2	**< 0.001**
CKD stage (n, %)				
1	425 (47.4)	19 (27.1)	406 (49.2)	**< 0.05**
2	194 (21.7)	22 (31.4)	172 (20.8)	
3	180 (20.1)	18 (25.7)	162 (19.6)	
4	97 (10.8)	11 (15.7)	86 (10.4)	
Hemoglobin (g/L), m ± sd	98.9 ± 20.66	84.46 ± 21.96	100.12 ± 23.44	**< 0.001**
RBC (10^12/L), m ± sd	3.52 ± 0.66	3.03 ± 0.78	3.56 ± 0.84	**< 0.001**
N (10^9/L), m ± sd	4.6 ± 3.21	3.92 ± 3.38	4.66 ± 3.13	**0.070**
L (10^9/L), m ± sd	1.43 ± 0.87	1.01 ± 0.56	1.47 ± 0.96	**< 0.001**
Serum creatinine (μmol/L), med (IQR)	83 (60.75, 133)	106 (76, 219.75)	81 (56.75, 152)	**0.001**
Uric acid (μmol/L), med (IQR)	392 (299.75, 503.25)	469 (337.50, 615.50)	387 (289.45, 515.50)	**0.005**
Serum urea nitrogen (mmol/L), m ± sd	10.33 ± 8.44	13.03 ± 9.62	10.1 ± 7.71	**0.007**
Serum albumin (g/L), med (IQR)	27 (21, 36)	28 (21, 32)	26 (22, 33)	0.338
Chol (mmol/L), m ± sd	6.12 ± 2.45	5.22 ± 1.87	6.2 ± 2.49	**< 0.001**
24 h proteinuria (g), med (IQR)	1.82 (0.695, 3.55)	2.4 (0.955, 3.7)	1.62 (0.755, 3.155)	0.180
Erythrocyturia (*n*, %)	688 (76.79)	59 (84.29)	629 (76.15)	0.122
leukocyturia (*n*, %)	333 (37.17)	23 (32.86)	310 (37.53)	0.437
Granular cast (*n*, %)	249 (27.79)	23 (32.86)	226 (27.36)	0.324
Anemia (*n*, %)	623 (69.53)	63 (90)	560 (67.8)	**< 0.001**
Leukopenia (*n*, %)	187 (20.87)	30 (42.86)	157 (19.01)	**< 0.001**
Anti‐cardiolipin IgM positivity (*n*/*N*, %)	145/655 (22.14)	24/51 (47.1)	121/604 (20)	**< 0.001**
Anti‐cardiolipin IgG positivity (*n*/*N*, %)	229/707 (32.4)	24/52 (46.2)	205/655 (31.3)	**0.002**
Positive ANA, (*n*, %)	848 (94.64)	68 (97.14)	780 (94.43)	0.333
Positive anti‐dsDNA, (*n*, %)	706 (78.79)	59 (84.29)	647 (78.33)	0.242
Positive anti‐Sm (*n*, %)	259 (28.91)	20 (28.57)	239 (28.93)	0.949
Positive anti‐SSA (*n*, %)	423 (47.21)	31 (44.29)	392 (47.46)	0.610
Positive anti‐SSB (*n*, %)	223 (24.89)	16 (22.86)	207 (25.06)	0.682
Positive anti‐RNP (*n*, %)	329 (36.72)	25 (35.71)	304 (36.8)	0.856
Positive anticentromere (n, %)	883 (98.55)	69 (98.57)	814 (98.55)	0.947
Complement C3 (g/L), m ± sd	0.63 ± 0.33	0.45 ± 0.34	0.64 ± 0.35	**< 0.001**
Complement C4 (g/L), m ± sd	0.16 ± 0.35	0.119 ± 0.34	0.163 ± 0.36	0.320
Hypocomplementemia (*n*, %)	592 (66.07)	57 (81.43)	535 (64.77)	**0.005**
SLE‐DAI	14.32 ± 4.66	15.56 ± 4.93	14.21 ± 5.24	**0.033**

*Note:* Data are expressed as mean ± standard deviation (m ± sd) and median (med) with interquartile range (IQR).

Abbreviations: ANA, antinuclear antibodies; CKD, chronic kidney disease; Chol, cholesterol; dsDNA, double‐stranded DNA; eGFR, estimated glomerular filtration rate; L, lymphocyte; N, neutrophil; RBC, red blood cell; RNP, ribonucleoprotein; SSA, sjögren syndrome antigen A; SSB, sjögren syndrome antigen B; SLE‐DAI, systemic lupus erythematosus disease activity index.

In terms of clinical manifestations, the incidence rate of oral ulcers in the thrombocytopenia group was higher than that in the control group (17.14% vs. 7.63%, *p* = 0.006). In addition, the thrombocytopenia group has higher SLE‐DAI score (15.56 ± 4.93 vs. 14.21 ± 5.24, *p* = 0.033).

Regarding laboratory parameters, LN patients with thrombocytopenia exhibited higher levels of serum creatinine (*p* = 0.001), urea nitrogen (*p* = 0.007), and uric acid (*p* = 0.005) compared to those without thrombocytopenia. The mean values of complement C3 and complement C4 were 0.45 ± 0.34 g/L and 0.11 ± 0.34 g/L, respectively, in LN patients with thrombocytopenia. LN patients with thrombocytopenia had higher frequency of hypocomplementemia (C3) (*p* = 0.005), anemia (*p* < 0.001) and leucopenia (*p* < 0.001). However, there was no significant difference in serum albumin, 24‐h proteinuria, microscopic red blood cells and microscopic white blood cells between the two groups. There was no significant difference in the proportion of positive antibodies between the two groups except for a higher proportion of positive anti‐cardiolipin antibodies in LN patients with thrombocytopenia.

### 3.2 Kidney Histopathological Characteristics of LN Patients With Thrombocytopenia and Without Thrombocytopenia

In this study, 774 LN patients underwent renal biopsy, including 56 in the thrombocytopenia group. The renal histopathological characteristics of LN patients with thrombocytopenia and without thrombocytopenia are shown in Table 2. LN patients with thrombocytopenia had higher activity index score (AI, *p* = 0.043), indicating more severe endocapillary hypercellularity (*p* = 0.025), medullary loop necrosis (*p* < 0.001), mesangial cell and matrix hyperplasia (*p* = 0.010), renal interstitial edema (*p* = 0.048). However, there was no significant difference in the chronicity index (CI) score between the two groups (*p* = 0.243). Additionally, comparable proportions of glomerular sclerosis, fibrous crescents, tubular atrophy, and interstitial fibrosis were observed between the two groups. Regarding drug treatment, more antiplatelet drugs were used in LN patients with thrombocytopenia (*p* < 0.001), while there was no significant difference between the two groups in other treatments. There was no significant difference in immunosuppressive therapy between the two groups (*p* = 0.231). We conducted renal biopsies on 774 patients and found that 20 of them exhibited thrombotic microangiopathy (TMA) changes. Among these, 2 were in the thrombocytopenia group, which was not significantly different from the group without thrombocytopenia (*p* = 0.963).

**Table 2:** Comparison of renal pathological characteristics and treatment methods in LN patients between the two groups.

Parameters	Total	With thrombocytopenia	Without thrombocytopenia	*p* values
Number of patients (*N*)	774	56	718	
Pathological classification of lupus nephritis (*n*, %)				
I	10 (1.29)	1 (1.79)	9 (1.25)	0.198
II	87 (11.24)	3 (5.36)	84 (11.7)	
III/IV/III + V/IV + V	521 (67.31)	42 (75.01)	479 (66.72)	
V	141 (18.22)	8 (14.29)	133 (18.52)	
VI	15 (1.94)	2 (3.57)	13 (1.81)	
Activity index, med (IQR)	6 (5–9)	6 (5–9)	6 (4–8)	**0.043**
Endocapillary hypercellularity (*n*, %)				
Focal segmental	343 (44.32)	26 (46.43)	317 (44)	**0.025**
Diffuse	214 (27.65)	19 (33.36)	197 (27)	
Mesangial cell and matrix hyperplasia (*n*, %)	434 (56.07)	40 (71.43)	394 (54.87)	**0.010**
Glomerular leukocyte infiltration (*n*, %)				
None	267 (34.5)	19 (33.93)	248 (34.54)	0.374
Light	310 (40.05)	24 (42.86)	286 (39.83)	
Moderate	169 (21.83)	9 (16.07)	160 (22.28)	
Severe	28 (3.62)	4 (7.14)	24 (3.34)	
Nuclear fragmentation (*n*, %)				
0	418 (54.01)	31 (55.36)	387 (53.9)	0.388
< 25%	285 (36.82)	17 (30.36)	268 (37.33)	
25%–50%	56 (7.24)	7 (12.5)	49 (6.82)	
> 50%	15 (1.94)	1 (1.79)	14 (1.95)	
Medullary loop necrosis (*n*, %)	79 (10.21)	10 (17.86)	69 (9.61)	**< 0.001**
Cellular crescents, med (IQR)	0 (0‐1)	0 (0‐1)	0 (0–1)	0.170
Platinum loop (n, %)	520 (67.18)	38 (67.86)	482 (67.13)	0.911
Vascular hypertrophy (*n*, %)	558 (72.09)	42 (75)	516 (71.87)	0.612
Interstitial inflammation (*n*, %)				
0	195 (25.19)	14 (25)	181 (25.21)	0.688
＜ 25%	453 (58.53)	29 (51.79)	424 (59.05)	
25%–50%	88 (11.37)	9 (16.07)	79 (11)	
50%–75%	28 (3.62)	3 (5.36)	25 (3.48)	
＞ 75%	10 (1.29)	1 (1.79)	9 (1.25)	
Renal interstitial edema (*n*, %)	157 (20.28)	17 (30.36)	140 (19.5)	**0.048**
Chronicity index, med (IQR)	3 (2–5)	3 (2–5)	3 (2–4)	0.243
Glomerular sclerosis, med (IQR)	0 (0–3)	0 (0–2)	0 (0–3)	0.530
Fibrous crescents, med (IQR)	0 (0‐0)	0 (0‐0)	0 (0‐0)	0.314
Tubular atrophy (*n*, %)				
0	321 (41.47)	28 (50)	293 (40.81)	0.129
＜ 25%	355 (45.87)	17 (30.36)	338 (47.08)	
25%–50%	77 (9.95)	9 (16.07)	68 (9.47)	
50%–75%	18 (2.33)	2 (3.57)	16 (2.23)	
＞ 75%	3 (0.39)	0 (0)	3 (0.42)	
Interstitial fibrosis (*n*, %)				
0	323 (41.73)	22 (39.29)	301 (41.92)	0.246
＜ 25%	361 (46.64)	25 (44.64)	336 (46.8)	
25%–50%	64 (8.27)	5 (8.93)	59 (8.22)	
50%–75%	17 (2.2)	2 (3.57)	15 (2.09)	
＞ 75%	7 (0.9)	2 (3.57)	5 (0.7)	
Glucocorticoids (*n*, %)	875 (97.66)	67 (95.71)	808 (97.82)	0.263
Immunosuppressive (*n*, %)	551 (61.5)	47 (67.14)	504 (61.02)	0.312
Antiplatelet drugs (*n*, %)	298 (33.26)	30 (42.86)	268 (32.45)	**< 0.001**
Treatment (*n*, %)				
GC + CTX	221 (24.7)	16 (22.9)	205 (24.8)	0.308
GC + MMF	93 (10.4)	3 (4.3)	90 (10.9)	
GC + CNI	14 (1.6)	1 (1.4)	13 (1.6)	
Others	568 (63.4)	50 (71.4)	518 (62.7)	
Systolic, med (IQR)	125 (113–140)	131 (110–140)	126 (113–140)	0.42
Diastolic, med (IQR)	80 (70–90)	84 (75–96)	80 (70–90)	0.739
Renal TMA	20/774 (2.6)	2/56 (3.6)	18/718 (2.6)	0.963

*Note:* Data are expressed as mean ± standard deviation (m ± sd) and median (med) with interquartile range (IQR).

Abbreviations: CNI, calcineurin inhibitor; CTX, cyclophosphamide; GC, glucocorticoid; MMF, mycophenolate mofetil; TMA, thrombotic microangiopathy.

As shown in Supporting Information Table S2, the variance inflation factor (VIF) has used to explain the independent relationship of several factors in the model. VIF < 10 indicates that several factors in the model are independent of each other. Leucopenia (*p* = 0.001), eGFR (*p* = 0.014) and anti‐cardiolipin IgM positivity (*p* < 0.001) were independent related factors associated with thrombocytopenia in LN patients. Multivariate binary logistic regression analysis revealed no significant association between the activity index and LN with thrombocytopenia, as shown in Table 3.

**Table 3:** Multivariable binary logistic regression analysis on related factors of LN with thrombocytopenia.

Multivariable binary logistic regression analysis	OR	Odds ratio (95% CI)	*p* value
Age	1.006	0.978–1.034	1.006
Gender (female)	0.851	0.290–2.500	0.769
Oral ulcer	1.401	0.743–2.641	0.297
eGFR	0.990	0.982–0.998	**0.014**
24 h proteinuria	0.982	0.858–1.124	0.792
Leukopenia	3.050	1.605–5.796	**0.001**
Hypocomplementemia	1.981	0.882–4.449	0.098
Anemia	2.595	0.935–7.205	0.067
Anti‐cardiolipin IgM positivity	3.891	2.081–7.275	**< 0.001**
Activity index	0.982	0.858–1.124	0.903

### 3.3 Patient Overall Survival and Kidney Survival of LN Patients With Thrombocytopenia and Without Thrombocytopenia

**Figure 1:**
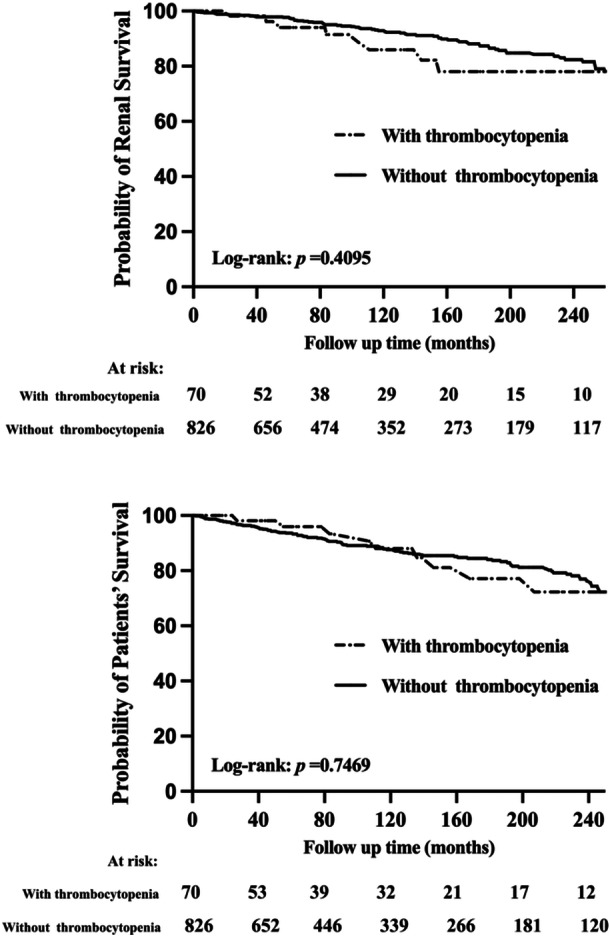
Renal and patient' survival of LN patients with and without thrombocytopenia.

In LN patients with thrombocytopenia, 10 patients died, and 8 patients reached the adverse renal outcomes. In LN patients without thrombocytopenia, 99 patients died, and 83 patients reached the adverse renal outcomes. The median follow‐up time in our study was 121 months. There was no significant difference in renal survival (*p* = 0.410) and patient survival (*p* = 0.747) between the two groups, as shown in Figure 1. After adjusting for potential confounding factors, Cox regression analysis showed that the renal outcomes and mortality were not significantly different for LN patients with and without thrombocytopenia, as shown in Supporting Information Table S3.

### 3.4 Multivariate Cox Risk Regression Analysis of LN Patients With Thrombocytopenia

Univariate COX risk regression analysis of LN patients with thrombocytopenia showed that Age and eGFR were risk factors for adverse renal outcomes. Positive anti‐cardiolipin IgM and anemia were risk factors for patient mortality, as shown in Supporting Information Table S4. Multivariate Cox regression analysis in LN patients with thrombocytopenia showed that lower eGFR (HR 95%CI, *p* = 0.032) was a risk factor for adverse renal outcomes, and anemia (HR 95%CI, *p* = 0.038) was a risk factor for patient mortality, as shown in Table 4.

**Table 4:** Multivariate COX risk regression analysis of LN patients with thrombocytopenia.

Cox hazard regression analysis	Renal adverse outcomes		Mortality	
	HR (95%CI)	*p* value	HR (95%CI)	*p* value
Age (per 1‐year increase)	0.990 (0.966–1.014)	0.407	1.006 (0.986–1.027)	0.543
Gender (female)	0.887 (0.351–2.240)	0.800	1.157 (0.551–2.430)	0.699
Oral ulcer (+)	0.859 (0.502–1.471)	0.581	0.758 (0.470–1.221)	0.255
eGFR (per 1 mlL/min/1.73 m^2^ increase)	0.994 (0.989–1.000)	**0.032**	0.998 (0.993–1.004)	0.602
Urine protein (per 1 g/24 h increase)	1.060 (0.964–1.165)	0.228	0.992 (0.897–1.098)	0.882
Leukocytes (per 10^9^/L increase)	0.990 (0.540–1.816)	0.974	1.368 (0.842–2.222)	0.206
Complement 3 (per 0.1 g/L increase)	0.839 (0.485–1.452)	0.531	0.639 (0.405–1.008)	0.054
Anemia (+)	1.843 (0.901–3.769)	0.094	1.887 (1.035–3.441)	**0.038**
Anti‐cardiolipin IgM (+)	1.019 (0.575–1.808)	0.947	0.889 (0.531–1.489)	0.656
Activity index (per 1 point increase)	1.009 (0.956–1.064)	0.748	0.991 (0.946–1.037)	0.687

## 4 Discussion

To date, approximately 20 clinical retrospective cohort studies have investigated SLE with thrombocytopenia. Consistent with previous research, our findings confirm that LN patients with thrombocytopenia exhibit higher disease activity, increased anti‐cardiolipin antibody positivity, and a higher activity index in kidney biopsy results. However, unlike previous studies, our findings suggest that the prognosis of patients with LN and thrombocytopenia may be comparable to those without thrombocytopenia. In addition, our study provides new insights, indicating that anemia is a risk factor for mortality in LN patients with thrombocytopenia, while lower eGFR is associated with adverse renal outcomes. The clinical significance of these findings lies in highlighting the importance for clinicians to recognize that LN patients with thrombocytopenia tend to have more active disease. However, with appropriate treatment, their prognosis may not necessarily be worse.

In this study, among the 896 patients diagnosed with lupus nephritis (LN), a total of 70 individuals (7.8%) were found to have thrombocytopenia. Previous studies have reported varying prevalence rates of thrombocytopenia in patients with SLE, ranging from 16.3% to 22.1% [5, 6, 7]. In our study, 7.8% of patients were diagnosed with thrombocytopenia. Our results showed that the prevalence of thrombocytopenia is lower in the LN cohort. This lower prevalence might be attributed to the inclusion of hospitalized patients only, as some individuals with thrombocytopenia may receive treatment in outpatient clinics. Moreover, a potential selection bias exists in our cohort, as most LN patients underwent renal biopsy, which is typically avoided in individuals with severe thrombocytopenia.

Thrombocytopenia observed in SLE patients is a sign of disease deterioration and poor prognosis [6, 20]. SLE patients presenting with thrombocytopenia often manifest more severe manifestations of the disease, including renal involvement [21], hematologic manifestations (primarily hemolytic anemia and leukopenia), and immunologic manifestations (antiphospholipid antibody) [22, 23, 24]. In our study, we observed that LN patients with thrombocytopenia exhibited higher SLE‐DAI scores. Prior investigations have reported that approximately 50‐60% of SLE patients suffer from leukopenia [8], which is an important risk factor affecting the renal prognosis of SLE patients [7]. In our study, we identified leukopenia as an independent factor associated with the occurrence of thrombocytopenia in LN patients.

Antiphospholipid syndrome (APS) is widely recognized to be associated with thrombocytopenia [25, 26]. In patients with systemic lupus erythematosus (SLE) and related autoimmune disorders, it is believed that antiphospholipid antibodies play a role in platelet destruction [27]. It has been reported that thrombocytopenia has a strong correlation with the increase of anti‐cardiolipin antibody IgM positivity and anti‐cardiolipin antibody IgG positivity levels in SLE [27]. Notably, in a cohort of South African patients [5], it has been observed that thrombocytopenia was prevalent among individuals with lupus nephritis (LN) who tested positive for anti‐cardiolipin antibodies. In our study, we found significantly higher rates of anti‐cardiolipin IgM antibody and anti‐cardiolipin IgG antibody positivity in LN patients with thrombocytopenia, aligning with the findings of previously published studies.

LN patients with thrombocytopenia had lower eGFR compared to those without thrombocytopenia. However, there are few studies including renal biopsies on LN patients with thrombocytopenia at present. Our results indicated that LN patients suffering from thrombocytopenia possessed a higher activity index in renal biopsy, which was comprised of more severe endocapillary hypercellularity, medullary loop necrosis, mesangial cell, as well as matrix hyperplasia.

Our study found a higher prevalence of anti‐cardiolipin antibodies in LN with thrombocytopenia patients. This study did not assess bleeding or thrombotic events. Further investigations are warranted to explore the appropriateness and effectiveness of antiplatelet therapy in LN patients with thrombocytopenia.

The median follow‐up of the 896 LN patients was about 121 months. The prognosis was similar between LN patients with and without thrombocytopenia, while the previous cohort reported lower survival rates for SLE patients with thrombocytopenia [11]. The above discrepancy may be attributed to differences in patient selection criteria. Additionally, it is noteworthy that our investigation documented a low incidence of severe thrombocytopenia among the LN patient population.

Lower eGFR contributes to the prediction of poor kidney prognosis in LN patients, and our findings align with similar results in the literature [16, 28, 29]. In addition, anemia was a risk factor for death in LN patients with thrombocytopenia. According to prior research [30], there is a correlation between autoimmune hemolytic anemia (AIHA) and thrombocytopenia in individuals with SLE. Moreover, AIHA patients in this population tend to exhibit a reduced survival rate. Interestingly, previous reports have shown that patients with positive antiphospholipid antibodies have a higher proportion of hemolytic anemia, especially IgM antibodies β 2 Glycoprotein I [31]. In our study, there may be concurrent lupus‐induced hemolytic anemia or microangiopathy‐induced hemolysis, as well as some cases of renal anemia. However, it is difficult for us to differentiate between these conditions, and therefore, further research is needed.

The present study has certain limitations that should be acknowledged. Firstly, this investigation was conducted as a single‐center retrospective analysis, which may affect the generalizability of the finding. We need further study with a large sample and multi‐center. Secondly, data of thromboembolic events or bleeding‐related events were not available for analysis. Furthermore, several tests, such as those for platelet antibodies, a disintegrin and metalloproteinase with thrombospondin motifs 13 (ADAMTS13), ADAMTS13 antibodies, complement genes, and bone marrow puncture, were not performed uniformly across all patients. As a result, the direct association between the observed thrombocytopenia in these individuals and lupus‐related damage to the hematological system remains uncertain. In addition, the diagnosis of thrombocytopenia occurred at admission, and we did not consider platelet changes during follow‐up, which is also a limitation of this study. Finally, our study only collected the initial treatments. Since patients may switch to different treatments during the follow‐up process, such differences will affect the analysis results, which is indeed a shortcoming of our study. The causes of thrombocytopenia in lupus are highly diverse and exhibit considerable heterogeneity. Therefore, our study population exhibits certain heterogeneity, which may impact the results. Although there was no significant difference in the incidence of TMA changes in renal biopsy pathology between patients with thrombocytopenia and those without TP in the study, our research is limited by the availability of only renal pathology data regarding TMA and the lack of laboratory results related to microangiopathic hemolytic anemia (MAHA). This precludes detailed identification and assessment of systemic TMA in patients, which is a limitation of our study. A comprehensive analysis of data pertinent to prognosis remains unexplored, presenting a future research avenue.

## 5 Conclusions

In summary, LN patients with thrombocytopenia showed higher SLE‐DAI, more anti‐cardiolipin antibody positivity and higher AI in kidney biopsy compared to those without thrombocytopenia. But the prognosis was similar between these two groups. Lower eGFR was a risk factor for adverse renal outcomes and anemia was a risk factor for death for LN patients with thrombocytopenia. A larger‐scale multicenter prospective study is needed to further explore the role and mechanism of thrombocytopenia in LN.

## Author Contributions

**Hui Diao:** conceptualization, data curation, formal analysis, methodology, resources, writing – original draft. **Yuting Fan:** data curation, formal analysis, methodology, resources, writing – original draft. **Di Kang:** data curation, investigation, resources. **Zhiqing Chen:** data curation, investigation, resources. **Yuewen Lu:** data curation, investigation, resources. **Xiamin Huang:** data curation, investigation, resources. **Xi Xia:** conceptualization, methodology, project administration, writing – review & editing. **Wei Chen:** conceptualization, funding acquisition, project administration, writing – review and editing.

## Ethics Statement

This study was performed in line with the principles of the Declaration of Helsinki. Approval was granted by the First Affiliated Hospital of Sun Yat‐sen University (December 13, 2016/Ethical review NO. [2016]215).

## Consent

Informed consent was obtained from all individual participants included in the study. Informed consent was obtained from all subjects involved in the study. Written informed consent has been obtained from the patients to publish this paper.

## Conflicts of Interest

The authors declare no conflicts of interest.

## Supporting information

Figure_1_SuppInfo Recruitment and exclusion flowchart.

Supporting information.

Supporting information.

Supporting information.

## Data Availability

The datasets generated during and/or analyzed during the current study are available from the corresponding author on reasonable request.
