# Supplementary figures and images for: Clinicopathological Characteristics and Outcomes of Lupus Nephritis Patients With Thrombocytopenia: A Single‐Center Retrospective Study
Source: Immun Inflamm Dis. 2025 Mar 19;13(3):e70179. doi: 10.1002/iid3.70179 (PMC11921463; doi:10.1002/iid3.70179)

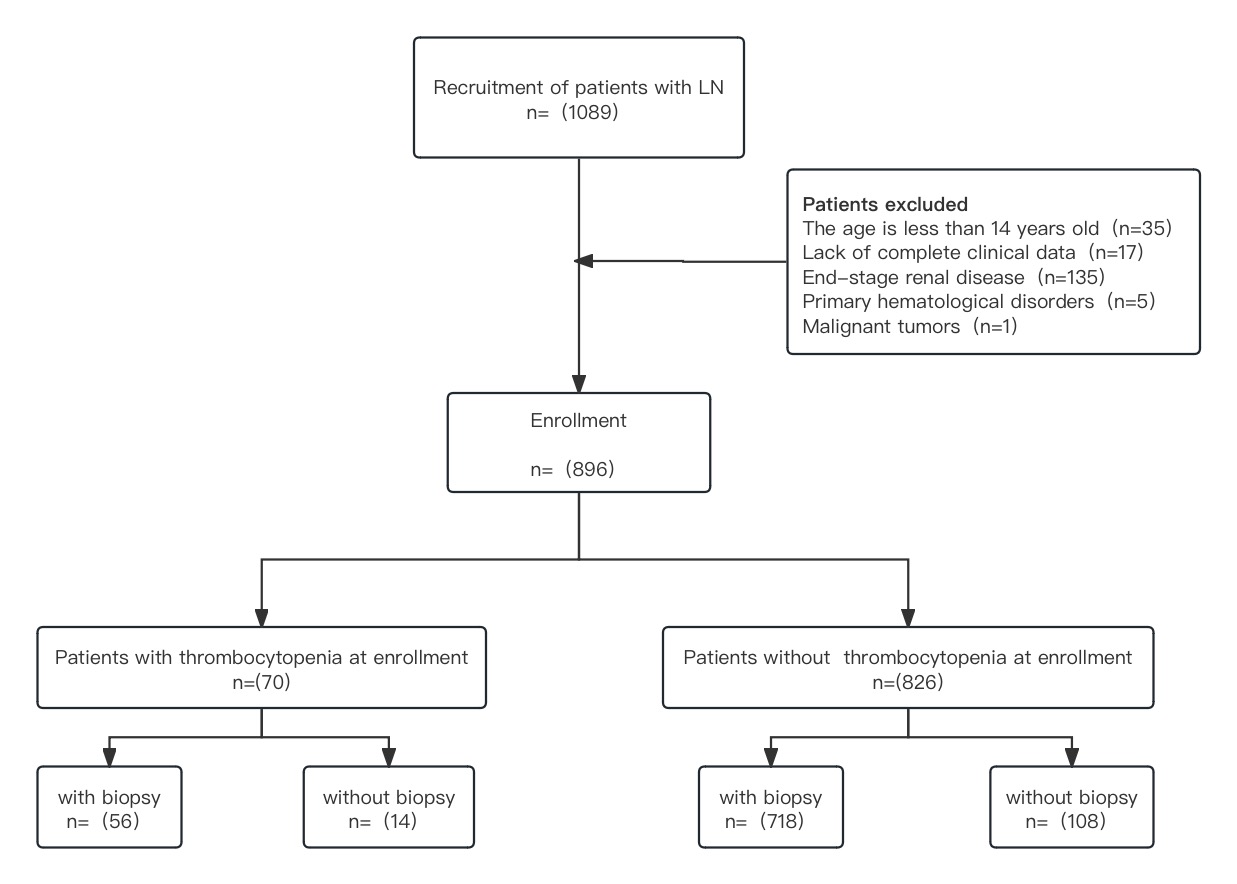

Supplement: Supplementary file 1 — Figure_1_SuppInfo Recruitment and exclusion flowchart. [file IID3-13-e70179-s002.jpg]
