# Supplementary material for: Clinicopathological Characteristics and Outcomes of Lupus Nephritis Patients With Thrombocytopenia: A Single‐Center Retrospective Study
Source: Immun Inflamm Dis. 2025 Mar 19;13(3):e70179. doi: 10.1002/iid3.70179 (PMC11921463; doi:10.1002/iid3.70179)
Supplement: Supplementary file 2 — Supporting information. [file IID3-13-e70179-s001.docx]

**Table_2_SuppInfo** Collinear statistical analysis of independent relationships among factors in the model

| Model | Collinear Statistics | |
| --- | --- | --- |
|  | **Tolerance** | **VIF** |
| Age | 0.850 | 1.176 |
| Gender(female) | 0.970 | 1.030 |
| Oral ulcer | 0.986 | 1.014 |
| eGFR | 0.735 | 1.361 |
| 24 hours proteinuria | 0.953 | 1.050 |
| Leukopenia | 0.957 | 1.045 |
| Hypocomplementemia | 0.947 | 1.056 |
| Anemia | 0.847 | 1.180 |
| Anti-cardiolipin IgM positivity | 0.961 | 1.040 |
| Activity index | 0.932 | 1.022 |

Note: VIF, variance inflation factor.
