# Supplementary material for: Clinicopathological Characteristics and Outcomes of Lupus Nephritis Patients With Thrombocytopenia: A Single‐Center Retrospective Study
Source: Immun Inflamm Dis. 2025 Mar 19;13(3):e70179. doi: 10.1002/iid3.70179 (PMC11921463; doi:10.1002/iid3.70179)
Supplement: Supplementary file 3 — Supporting information. [file IID3-13-e70179-s004.docx]

**Table_3_SuppInfo** Multivariate COX risk regression analysis between LN patients with and without thrombocytopenia

| Cox hazard regression analysis | Renal adverse outcome | | | Mortality | | |
| --- | --- | --- | --- | --- | --- | --- |
|  | **HR (95%CI)** | ***P* value** | **HR (95%CI)** | | ***P* value** |  |
| Unadjusted risk | 1.255 (0.607 - 2.595) | 0.540 | 1.189 (0.653 - 2.163) | | 0.571 |  |
| Model 1 | 1.252 (0.605 - 2.592) | 0.544 | 1.201 (0.660 - 2.186) | | 0.549 |  |
| Model 2 | 1.179 (0.567 - 2.448) | 0.660 | 1.085 (0.578 - 2.038) | | 0.800 |  |
| Model 3 | 1.324 (0.627 - 2.798) | 0.462 | 1.128 (0.611 - 2.083) | | 0.701 |  |
| Model 4 | 1.505 (0.671 - 3.378) | 0.321 | 1.226 (0.606 - 2.481) | | 0.570 |  |

Model 1 was adjusted for age, gender.

Model 2 was adjusted for age, gender, eGFR, 24 hours proteinuria.

Model 3 was adjusted for age, gender, leukopenia, hypocomplementemia, Oral ulcer.

Model 4 was adjusted for age, gender, Oral ulcer, hypocomplementemia, eGFR, 24 hours proteinuria, anemia, anti-cardiolipin IgM positivity, activity index.
