# Supplementary material for: Clinicopathological Characteristics and Outcomes of Lupus Nephritis Patients With Thrombocytopenia: A Single‐Center Retrospective Study
Source: Immun Inflamm Dis. 2025 Mar 19;13(3):e70179. doi: 10.1002/iid3.70179 (PMC11921463; doi:10.1002/iid3.70179)
Supplement: Supplementary file 4 — Supporting information. [file IID3-13-e70179-s003.docx]

**Table_4_SuppInfo** Univariate COX risk regression analysis of LN patients with thrombocytopenia

| **Cox hazard regression analysis** | **Renal adverse outcome** | | **Mortality** | |
| --- | --- | --- | --- | --- |
|  | **HR (95%CI)** | **P value** | **HR (95%CI)** | **P value** |
| Age  (per 1 year increase) | 2.621 (0.723 - 12.836) | **0.038** | 1.024 (0.952 - 1.101) | 0.576 |
| Gender  (female) | 1.282 (0.083 - 20.628) | 0.923 | 1.002 (0.985 - 1.019) | 0.069 |
| Fever  (+) | 1.436 (0.976 - 5.284) | 0.085 | 6.024 (0.329 - 10.994) | 0.077 |
| Oral ulcer  (+) | 4.721 (0.090 - 8.642) | 0.078 | 1.090 (0.507 - 2.346) | 0.770 |
| Alopecia  (+) | 1.031 (0.314 - 1.846) | 0.730 | 1.071 (0.980 - 1.170) | 0.386 |
| Leukocytes  (per 109/L increase) | 0.613 (0.061 - 5.632) | 0.590 | 2.742 (0.035 - 3.462) | 0.231 |
| Anemia  (+) | 2.942 (0.937 - 12.642) | 0.068 | 1.634 (0.326 - 2.743) | **0.002** |
| Complement C3  (per 0.1 g/L increase) | 0.952 (0.0842 - 3.314) | 0.069 | 0.992 (0.078 - 1.109) | 0.195 |
| Anti-cardiolipin  IgG (+) | 1.264 (0.032 - 32.471) | 0.998 | 0.350 (0.064 - 1.910） | 0.988 |
| Anti-cardiolipin  IgM (+) | 2.314 (1.425 - 8.361) | 0.056 | 1.056 (0.024 - 4.271) | 0.046 |
| Urine protein (per 1 g/24 hours increase) | 1.052 (0.315 - 1.732) | 0.383 | 6.145 (0.871 - 28.132) | 0.226 |
| Serum creatinine  (per 100 μmol/L increase) | 1.145 (0.743 - 2.849) | 0.892 | 2.731 (0.981 - 4.271) | 0.078 |
| Serum urea nitrogen  (per 1mmol/L increase) | 9.121 (1.842 - 38.841) | 0.901 | 1.001 (0.007 - 1.076) | 0.815 |
| eGFR (per 1 ml/min/1.73m2 increase) | 0.834 (0.065 - 2.846) | 0.019 | 0.738 (0.01 - 1.836) | 0.083 |
| Uric acid  (per 1μmol/L increase) | 22.731 (0.001 - 261.842) | 0.059 | 1.003 (0.076 - 2.843) | 0.191 |
| SLE-DAI  (per 1 increase) | 1.081 (0.761 - 4.425) | 0.485 | 1.542 (0.438 - 1.964) | 0.190 |
| dsDNA  (+) | 1.912 (0.391 - 10.631) | 0.651 | 11.637 (0.55 - 36.729) | 0.244 |
| Renal interstitial edema (+) | 1.082 (0.021 - 3.923) | 0.883 | 1.276 (0.001 - 2.009) | 0.988 |
| Mesangial cell and matrix hyperplasia (+) | 1.483 (0.896 - 3.721) | 0.188 | 1.082 (0.660 - 1.890) | 0.085 |
| Activity index  (per 1 increase) | 1.142 (0.967 - 2.176) | 0.071 | 1.095 (0.091 - 2.093) | 0.178 |
